# Supplementary material for: Heterogeneity of synaptic connectivity in the fly visual system
Source: Nat Commun. 2024 Feb 21;15:1570. doi: 10.1038/s41467-024-45971-z (PMC10882054; doi:10.1038/s41467-024-45971-z)
Supplement: Supplementary file 3 — Description of Additional Supplementary Files [file 41467_2024_45971_MOESM3_ESM.pdf]

# Description of Additional Supplementary Files

**Supplementary Data 1. Contributions to the dataset used in this study.** This table contains a sheet in which all segment IDs, neuron types, and % edits are shown, as well as a sheet containing the neuron types and respective numbers annotated by each contributor, and the number of total annotations.
