# Supplementary material for: Heterogeneity of synaptic connectivity in the fly visual system
Source: Nat Commun. 2024 Feb 21;15:1570. doi: 10.1038/s41467-024-45971-z (PMC10882054; doi:10.1038/s41467-024-45971-z)
Supplement: Supplementary file 5 — Reporting Summary [file 41467_2024_45971_MOESM5_ESM.pdf]

nature portfolio

Corresponding author(s): *Double-anonymous peer review submissions: write DAPR and your manuscript number here instead of author names.*

Last updated by author(s): *YYYY-MM-DD*

Reporting Summary

Nature Portfolio wishes to improve the reproducibility of the work that we publish. This form provides structure for consistency and transparency in reporting. For further information on Nature Portfolio policies, see our Editorial Policies and the Editorial Policy Checklist.

Statistics

For all statistical analyses, confirm that the following items are present in the figure legend, table legend, main text, or Methods section.

n/a	Confirmed	
☐	☒	The exact sample size (*n*) for each experimental group/condition, given as a discrete number and unit of measurement
☐	☒	A statement on whether measurements were taken from distinct samples or whether the same sample was measured repeatedly
☐	☒	The statistical test(s) used AND whether they are one- or two-sided<br>*Only common tests should be described solely by name; describe more complex techniques in the Methods section.*
☐	☒	A description of all covariates tested
☐	☒	A description of any assumptions or corrections, such as tests of normality and adjustment for multiple comparisons
☐	☒	A full description of the statistical parameters including central tendency (e.g. means) or other basic estimates (e.g. regression coefficient) AND variation (e.g. standard deviation) or associated estimates of uncertainty (e.g. confidence intervals)
☐	☒	For null hypothesis testing, the test statistic (e.g. *F*, *t*, *r*) with confidence intervals, effect sizes, degrees of freedom and *P* value noted<br>*Give P values as exact values whenever suitable.*
☒	☐	For Bayesian analysis, information on the choice of priors and Markov chain Monte Carlo settings
☒	☐	For hierarchical and complex designs, identification of the appropriate level for tests and full reporting of outcomes
☐	☒	Estimates of effect sizes (e.g. Cohen's *d*, Pearson's *r*), indicating how they were calculated

Our web collection on statistics for biologists contains articles on many of the points above.

Software and code

Policy information about availability of computer code

Data collection	Leica LASX was used for ExM and Bruker Prairie View for 2P imaging data acquision, Flywire.ai for connectomics work.
Data analysis	Preprocessing of ExM data was done by utilizing the VVDViewer (https://github.com/JaneliaSciComp/VVDViewer).<br>Custom code in Matlab 2017b and Python 3.9 was used for data analysis and plotting. The code is available on GitHub: https://github.com/silieslab/Cornean_Molina-Obando_etal_2024.git<br>Python tools:<br>Anaconda, Visual Studio Code and Atom<br>EM Python packages:<br>numpy== 1.21.5, pandas==1.3.5, matplotlib==3.5.1, seaborn ==0.11.2,scipy=1.7.3, sklearn==1.0.2, statsmodels==0.13,5<br>ExM Python packages:<br>pyclesperanto-prototype==0.24.1, napari[pyqt5], PyQt5==5.15.9, napari-pyclesperanto-assistant==0.22.1, pydantic==1.10.9, typing-extensions==4.6.3, napari-roi==0.1.8, jupyterlab==4.0.9, pynrrd==1.0.0, seaborn==0.12.2, pandas==2.0.2, numpy==1.24.4, matplotlib==3.7.1<br>2P Matlab: standard toolboxes, as well as signal processing, image processing, statistics and machine learning toolboxes

For manuscripts utilizing custom algorithms or software that are central to the research but not yet described in published literature, software must be made available to editors and reviewers. We strongly encourage code deposition in a community repository (e.g. GitHub). See the Nature Portfolio guidelines for submitting code & software for further information.

Data

Policy information about availability of data

All manuscripts must include a data availability statement. This statement should provide the following information, where applicable:

- Accession codes, unique identifiers, or web links for publicly available datasets
- A description of any restrictions on data availability
- For clinical datasets or third party data, please ensure that the statement adheres to our policy

Source data of this study can be found on Zenodo https://zenodo.org/doi/10.5281/zenodo.10361474.

Research involving human participants, their data, or biological material

Policy information about studies with human participants or human data. See also policy information about sex, gender (identity/presentation), and sexual orientation and race, ethnicity and racism.

Reporting on sex and gender	This study did not involve human participants.
Reporting on race, ethnicity, or other socially relevant groupings	This study did not involve human participants.
Population characteristics	This study did not involve human participants.
Recruitment	This study did not involve human participants.
Ethics oversight	This study did not involve human participants.

Note that full information on the approval of the study protocol must also be provided in the manuscript.

Field-specific reporting

Please select the one below that is the best fit for your research. If you are not sure, read the appropriate sections before making your selection.

☒ Life sciences ☐ Behavioural & social sciences ☐ Ecological, evolutionary & environmental sciences

For a reference copy of the document with all sections, see nature.com/documents/nr-reporting-summary-flat.pdf

Life sciences study design

All studies must disclose on these points even when the disclosure is negative.

Sample size	For microscope data, sample sizes were chosen based on previous literature sample sizes (e.g. Ketkar et al. 2020 CurrBioo, Serbe et al. 2016 Neuron, Strother et al. 2017 Neuron).<br>The connectomics data comes from one single individuum, and 320 columns were analyzed. We did not pre-calculate sample size, and previous analysis of "variability" in the connectome was done on seven columns (Shinomiya et al. 2015 PNAS). We extended this analysis (for Tm9 connectivity) more than 40-fold, by analyzing 320 columns.
Data exclusions	For 2-photon microscopy no data was excluded for analysis, except when recordings were aborted due to strong motion of the living animal.<br>For confocal microscopy no data was excluded for analysis, unless the brain wasn't oriented properly for ExM.<br>For connectomics data, connections with less than 3 synaptic contacts were excluded, as well as columns in a damaged region of the brain.
Replication	Does not apply to the connectomics dataset (one fly), but for example, left and right optic lobes were analyzed separately and by different researchers, and no significant differences were founds. Furthermore, the same neuron types were annotated by different researchers in many instances, and their identity was later confirmed by J.C., M.S., or S.M.-O., such that identification of cell type identity was performed at least twice independently for each cell. Confocal analysis was done one n=7 brains, and this dataset was replicated in the lab on a different day, and characterization was also independently done by the FlyLight team (Janelia, https://flweb.janelia.org/cgi-bin/flew.cgi)
Randomization	For 2-photon and confocal microscopy, experimental groups were determined by the genotype of the flies, flies of different genotypes were recorded at similar time frames using the same experimental protocol. Stimulus presentation trials were pseudo-randomized.
Blinding	The investigators were not blinded to the fly genotypes, and the majority of the analysis presented here is done on a single wildtype fly, the same experimental procedure applied to all groups within a dataset.

Reporting for specific materials, systems and methods

We require information from authors about some types of materials, experimental systems and methods used in many studies. Here, indicate whether each material, system or method listed is relevant to your study. If you are not sure if a list item applies to your research, read the appropriate section before selecting a response.

Materials & experimental systems

n/a	Involved in the study
☐	☒ Antibodies
☒	☐ Eukaryotic cell lines
☒	☐ Palaeontology and archaeology
☐	☒ Animals and other organisms
☒	☐ Clinical data
☒	☐ Dual use research of concern
☒	☐ Plants

Methods

n/a	Involved in the study
☒	☐ ChIP-seq
☒	☐ Flow cytometry
☒	☐ MRI-based neuroimaging

Antibodies

Antibodies used

chicken anti-GFP, Abcam #13970, rabbit anti-DsRed, Takara Bio Clontech #632496, goat anti-chicken Alexa Fluor 488, Jackson Immunoresearch #103-545-155, goat anti-rabbit ATTO 647N, Sigma #40839

Validation

Abcam #13970 was validated in ICC (https://www.abcam.com/products/primary-antibodies/gfp-antibody-ab13970.html?productWallTab=Questions).
Takara Bio Clontech #632496, was validated by Western Blot (https://www.takarabio.com/documents/Certificate%20of%20Analysis/632543/632543-021313.pdf).

Animals and other research organisms

Policy information about studies involving animals; ARRIVE guidelines recommended for reporting animal research, and Sex and Gender in Research

Laboratory animals

Drosophila melanogaster females from 1 to 9 days post eclosion were used in this study.
Strains:
w+;Tm9[24C08]-LexAp65[attP40]. UAS-brp[short]::mCherry/ Tm16[82C05]-AD[attP40]; LexAop-rCD2::GFP/Tm16[52D11]-DBD[attP2]
w+;Tm9[24C08]-LexAp65[attP40]. UAS-brp[short]::mCherry/ Dm12-AD[attP40]; LexAop-rCD2::GFP/Dm12-DBD[attP2]
Dm12-split-Gal4 (SS00359, Aljoscha Nern, Janelia Research campus)
w+;Tm9[24C08]-LexAp65[attP40]. UAS-brp[short]::mCherry/ C3[R26H02]-AD; LexAop-rCD2::GFP/C3[R29G11]-DBD
w/w+;Tm924C08-LexA,lexAop-GCaMP6f/+;+/+
w+, norpA; Tm9[24C08]-LexA,LexAop-GCaMP6f/+;L3[0595]/+
w+, norpA; Tm9[24C08]-LexA,LexAop-GCaMP6f/+;Tm1[27b]/+
w+, norpA; Tm9[24C08]-LexA,LexAop-GCaMP6f/R35A03-AD;R29G11-DBD/+

Wild animals

The study did not involve wild animals.

Reporting on sex

The FAFB dataset is an EM reconstruction of one female fly. Thus, connectome analysis was complemented by also using female flies in confocal / ExM analysis. There is no known relevance of sex to the subject under investigation here / there is no known sexual dimorphism in peripheral visual circuitry of the fly eye. Differentiation of sex was done by the sex organs, shape and colour of the abdomen.

Field-collected samples

The study did not involve field-collected samples.

Ethics oversight

No ethical approval was required for fly experiments, the lab has all the permits to work with genetically modified organisms.

Note that full information on the approval of the study protocol must also be provided in the manuscript.

Plants

Seed stocks

The study did not involve seed stocks.

Novel plant genotypes

The study did not involve novel plant genotypes.

Authentication

The study did not involve plants.
